# Plasmid diversity, antibiotic resistance and virulence genes associated with *Staphylococcus aureus* isolates from private hospitals in Gauteng, South Africa

**DOI:** 10.1007/s11274-026-04956-4

**Published:** 2026-04-25

**Authors:** Kazadi Ngoie, Chanel Kingsburgh, Thabo Hamiwe, Marthie Ehlers

**Affiliations:** 1 Department of Medical Microbiology, Faculty of Health Sciences, University of Pretoria, Private Bag X323, Pretoria, Arcadia 0007 South Africa; 2 National Health Laboratory Service, Tshwane Academic Division, Pretoria, South Africa; 3 Department of Medical Microbiology, Ampath Laboratories, Centurion, South Africa

**Keywords:** Antibiotic resistance, Plasmids, Replicons, Sequence types, *Staphylococcus aureus*, Virulence genes

## Abstract

This study investigated the association between plasmid diversity, antibiotic resistance and virulence genes of *Staphylococcus aureus* (*S. aureus*) isolates from private hospitals in Gauteng, South Africa. Automated antibiotic susceptibility testing was done on 100 S. aureus isolates. Plasmid extraction and polymerase chain reaction (PCR)-based replicon typing were done on all isolates, followed by a multiplex-PCR assay screening for virulence genes in plasmid-positive isolates. Whole genome sequencing (WGS) was conducted on ten representative isolates selected based on the presence and number of plasmids, replicon gene profiles and antibiotic susceptibility patterns. Resistance to erythromycin (29%; 29/100), clindamycin (26%; 26/100) and ciprofloxacin (15%; 15/100) was observed. Plasmids were detected in 60% (60/100) of isolates, with pN315 (70%; 7/10) and pSaaS6159 (50%; 5/10) predominating among representative isolates and carrying antibiotic resistance genes (ARGs). Specific rep–ARG associations included rep7a (pS0385p1) with tetK, rep7a (pTZ4) with cat, rep10a (pDLK1) with ermC and rep5a (pN315) and rep16 (pSaaS6159) with blaZ. No virulence gene was specifically linked to any rep gene. Sequence types identified by WGS included ST22, ST35 and ST5 among MRSA isolates and ST152, ST5, ST6, ST1 and ST45 among MSSA isolates. These findings highlight an association between specific plasmids (pS0385p1, pTZ4, pDLK1, pN315 and pSaaS6159) and ARGs. The lack of association between replicon type and virulence gene carriage suggests that plasmid-borne traits in S. aureus are more strongly driven by antimicrobial selection than by virulence factors, with most virulence genes residing on the chromosome. Enhanced surveillance programs targeting these plasmids may improve understanding of ARG dissemination in healthcare settings.

**Supplementary Information:**

The online version contains supplementary material available at 10.1007/s11274-026-04956-4.

## Introduction

*Staphylococcus aureus* (*S. aureus*) is considered the most pathogenic species of the genus *Staphylococcus*, causing a wide range of diseases from superficial skin abscesses to severe, life-threatening conditions such as bacteraemia, endocarditis and necrotising pneumonia (Bitrus et al. 2018). *S. aureus* is one of the World Health Organization (WHO)-2017 designated ESKAPE (*Enterococcus faecium*,* methicillin-resistant S. aureus* (*MRSA*), *Klebsiella pneumoniae*,* Acinetobacter baumannii*,* Pseudomonas aeruginosa* and *Enterobacter species*) pathogens, a group of bacteria identified as leading causes of life-threatening infections (Sati et al. 2025). These bacteria are also notable for their ability to rapidly acquire and disseminate antibiotic resistance, which presents significant public health implications (Khasapane et al. 2024). Antibiotic resistance in *S. aureus* often involves the acquisition of mobile genetic elements (MGEs), such as plasmids that may be associated with the dissemination of antimicrobial resistance genes (ARGs) and virulence genes through horizontal gene transfer (HGT) (Foster 2017; Mores et al. 2021). These genetic elements contribute to bacterial adaptation and survival under antimicrobial pressure (Kumavath et al. 2025). For example, plasmids in *S. aureus* commonly harbour genes such as *blaZ* (β-lactam resistance), *ermC* (macrolide–lincosamide–streptogramin resistance), *tetK* (tetracycline resistance) and *cat* (chloramphenicol resistance), which are often associated with specific plasmid types (McCarthy and Lindsay 2012). In addition, virulence factors, such as biofilm formation, immune evasion strategies and toxin production, worsen the severity of *S. aureus* infections (Thammavongsa et al. 2015; Muthukrishnan et al. 2019).

Staphylococcal plasmids range from 1 kilobase pair (kb) to 60 kb in size and frequently carry multiple ARGs, which confer resistance to various antibiotics, biocides, and heavy metals (Haaber et al. 2017; Kwong et al. 2017). Clinical *S. aureus* isolates may carry one or more multidrug resistance plasmids per isolate and the coexistence of different plasmids within a single isolate can enhance the adaptation and dissemination of *S. aureus* in diverse environments (Neyaz et al. 2020; Mores et al. 2021). Understanding the prevalence and diversity of plasmids is essential for grasping the epidemiology of ARGs and virulence genes in this bacterium (Shahkarami et al. 2013; Al-Trad et al. 2023). Staphylococcal plasmids display variation in replication initiation proteins (Rep), which form the basis for plasmid classification (Al-Trad et al. 2023). These plasmids can carry one or more of seven specific types of Rep families: PriCT_1, RepA_N, Rep_1, Rep_2, Rep_3, RepL, and Rep_trans; each encoded by specific replication initiation (*rep*) genes (Jensen et al. 2009; Lozano et al. 2012; Kwong et al. 2017; Al-Trad et al. 2023). Classification based on *rep* gene sequences provides insight into plasmid diversity and supports the tracking of resistance-associated elements (Jensen et al. 2009; Kwong et al. 2017).

Despite the clinical importance of plasmids, studies in South Africa have largely focused on antibiotic resistance patterns and plasmid profiling, with limited emphasis on the molecular characterisation of *rep* genes. Polymerase chain reaction (PCR)-based replicon typing (PBRT) has been successfully used to characterise plasmid *rep* genes in *S. aureus* (Mahajan et al. 2023), and offers a cost-effective approach for resource-limited settings. Therefore, this study aimed to evaluate the relationship between plasmid diversity, antibiotic resistance profiles, and virulence genes in *S. aureus* isolates from private hospitals in Gauteng, South Africa, using multiplex PCR and PBRT assays, complemented by WGS of selected isolates.

## Materials and methods

### Study setting, collection and sub-culture of *Staphylococcus aureus* isolates

This descriptive laboratory-based study was conducted at the Department of Medical Microbiology, University of Pretoria (UP) and approved by the Research Ethics Committee (REC) (Reference: 460/2023) of the Faculty of Health Sciences, UP. A total of 100 invasive *S. aureus* isolates were collected from a private diagnostic laboratory in Gauteng, South Africa, between May and August 2023. Demographic data (age, sex, specimen type, ward and hospital) were recorded. Species identification and antibiotic susceptibility testing (AST) were performed at the diagnostic laboratory using matrix-assisted laser desorption/ionisation time-of-flight mass spectrometry (MALDI-TOF MS) (Bruker Daltonics Inc., USA) and the VITEK^®^ 2 automated system (bioMérieux, France), respectively. The AST panel included: ceftaroline, ciprofloxacin, clindamycin, cloxacillin, co-trimoxazole, daptomycin, erythromycin, gentamicin, linezolid, rifampicin, tetracycline and vancomycin.

### Total genomic DNA extraction of *Staphylococcus aureus* isolates

Isolates stored at -20 °C (Samsung, Korea) were inoculated into 5 mL of Brain Heart Infusion (BHI) broth (LabM Limited, UK) and incubated at 37 °C, 180 rpm for 24 h (Stuart Orbital, UK). The DNA extraction followed the boiling method protocol (Yang et al. 2008) with modifications: 1 500 µL broth was centrifuged (Labnet, USA) at 5 000 *xg* for five minutes, the pellet was resuspended in 1 000 µL of 1× Phosphate Buffered Saline (PBS) (pH 7.2) (Merck, Germany) After three seconds of vortexing (Labnet, USA) and a further centrifugation step at 5 000 x*g* for five minutes, the supernatant was discarded and 50 µL of 1X PBS (pH 7.2) (Merck, Germany) was added. The suspension was incubated (AccublockTM Digital dry bath, Labnet, USA) at 95 °C for 15 min, followed by sonication (Transsonic 460, Elma, Germany) at 35 kHz for 15 min. A final centrifugation step (Labnet, USA) was performed at 13 500 x*g* for five minutes and 50 µL of the supernatant containing DNA was transferred to a sterile microcentrifuge tube (Greiner Bio-One, Austria) and stored at -20 °C in a freezer (Samsung, Korea) until needed.

### Species confirmation and polymerase chain reaction-based replicon typing of the *Staphylococcus aureus* isolates.

An M-PCR assay was employed to confirm the identity (ID) of *S. aureus* species and to detect MRSA by targeting the *Staphylococcus* genus-specific small subunit 16 S ribosomal ribonucleic acid (rRNA) gene (597 base pairs (bp) (Al-Talib et al. 2009), the *S. aureus* species-specific thermonuclease (*nuc*) gene (359 bp) (Hirotaki et al. 2011) and the methicillin resistance gene (*mecA*) gene (310 bp) (Deshmukh et al. 2021). The strain *S. aureus* subspecies *aureus* Rosenbach ATCC 12600™, was used as a positive control for the detection of the 16 S rRNA and *nuc* genes, while ATCC 24213™ served as the positive control for the *mecA* gene and nuclease-free water (NFW) (Promega, USA) was used as a negative control. Primer sequences and cycling conditions are listed in Table 1.

**Table 1:** Primer sequences and reaction conditions for the identification and detection of selected virulence genes among *Staphylococcus aureus* isolates using multiplex-PCR

Identification PCR					
Target gene		Primer (5’ to 3’)	Amplicon Size (bp)	Reaction condition	References
*Staphylococcus* genus 16S rRNA		F- GCAAGCGTTATCCGGATTT	597	95°C for 15 min (1 cycle), 94°C for 30 sec, 58°C for 3 min, 72°C for 1 min and 30 sec (30 cycles), 72°C for 2 min (1 cycle)	Al-Talib et al., 2009
		R- CTTAATGATGGCAACTAAGC			
*Staphylococcus aureus* species-specific thermonuclease (*nuc*)		F- TCGCTTGCTATGATTGTGG	359		Hirotaki et al., 2011
		R- GCCAATGTTCTACCATAGC			
Methicillin-resistance (*mecA*)		F- AAAATCGATGGTAAAGGTTGGC	310		Deshmukh et al., 2021
		R- AGTTCTGCAGTACCGGATTTGC			
*S. epidermidisspecies*-specific fragment		F-ATCAAAAAGTTGGCGAACCTTTTCA	124		Martineau et al., 2000
		R-CAAAAGAGCGTGGAGAAAAGTATCA			
Virulence genes					
Virulence factor	Target gene	Primer sequence (5’ to 3’)	Amplicon size (bp)	Reaction condition	References
Biofilm formation	*clfA*	F-TTAATCGGTTTTGGACTACTC	73	95°C for 15 min (1 cycle), 94°C for 30 sec, 52°C for 3 min, 72°C for 1 min and 30 sec (30 cycles), 72°C for 2 min (1 cycle)	Cao et al., 2023
		R-CTAACGCTACTTGAATCATT			
	*clfB*	F-ACAGTAGGTACCACATCAGT	63		
		R-AAGATTGCGTTGTATCGTTC			
Exfoliative toxin	*eta*	F-ACTGTAGGAGCTAGTGCATTTGT	190		Budzy´nska et al., 2021
		R-TGGATACTTTTGTCTATCTTTTTCATCAAC			
	*etb*	F-ACAAGCAAAAGAATACAGCG	226		Mohseni et al. 2018
		R-GTTTTTGGCTGCTTCTCTTG			
Haemolysin	*hlgA*	F-GATGCCCTAGTTGTTAAGATG	177		Cao et al., 2023
		R-TTTCCGCCGATATTATAGCC			
	*hlgB*	F-AACTCAGGCTTTGTGAAACC	139		
		R-CCAAATGTATAGCCTAAAGT			
	*hlgC*	F-GAATCTACAAACGTGAGTCA	174		
		R-TTTGACCTGATTCAGTGGC			
	*hla*	R-GGTAGTCATCACGAACTCGT	166		
		R- GCATCAACTGTATTGGATAGCAAAAGC			

The PBRT typing scheme was utilised for the screening of *rep* genes in all isolates according to the protocol described by Neyaz et al. (2020). Currently, 26 *rep* genes have been characterised in Gram-positive genera, including *Bacillus*, *Enterococcus* and *S. aureus* (Jensen et al. 2009; Neyaz et al. 2020). However, the PBRT typing scheme employed in this study included 20 of the most commonly found *rep* genes in *S. aureus* based on previous studies (Jensen et al. 2009; Neyaz et al. 2020). This selection was made to minimise the number of M-PCR assays required, thereby reducing both the cost and time of analysis. The 20 *rep* genes were categorised into five M-PCR assays based on annealing temperature and amplification product sizes, as detailed in Table 2.

**Table 2:** Primer sequences and reaction conditions for the detection of selected *rep* genes among *Staphylococcus aureus* isolates (Jensen et al., 2009; Neyaz et al., 2020)

Rep Family	Species of plasmid origin		Primer sequence (5’ to 3’)	Amplicon size (bp)	Reaction condition
Group 1					
1	*Enterococcus faecalis*		F-TCGCTCAATCACTACCAAGC	624	95°C for 15 min (1 cycle), 94°C for 30 sec, 56°C for 3 min, 72°C for 1 min and 30 sec (30 cycles), 72°C for 2 min (1 cycle)
			R-CTTGAACGAGTAAAGCCCTT		
6	*E. faecalis*		F-ACGAATGAAAGATAAAGGAGTAG	551	
			R-TAAATTCTAGTTTGGCAATCTTAT		
7b	*S. aureus*		F-CTAATAGCCGGTTAGACGCAC	729	
			R-GACGRGARTTTCTATGTAATTCTCC		
8	*E. faecalis*		F-TAGATACGACAAAAGAAGAATTACA	394	
			R-CCAATCATGTAATGTTACAACC		
Group 2					
2	*E. faecalis*		F-GAGAACCATCAAGGCGAAAT	630	
			R-ACCAGAATAAGCACTACGTACAATCT		
9	*E. faecalis*		F-GCTCGATCARTTTTCAGAAG	201	
			R-CGCAAACATTTGTCWATTTCTT		
10a	*Bacillus subtilis*		F-TATAAAGGCTCTCAGAGGCT	382	
			R-CCAAATTCGAGTAAGAGGTA		
18	*E. faecalis*		F-ACACCAGTCGAAATGAATTT	462	
			R-AGGAATATCAAGTAATTCATGAAAGT		
Group 3					
3		*Bacillus thuringiensis*	F-CCTAATGTATATAATTTTGGTACATAT	403	95°C for 15 min (1 cycle), 94°C for 30 sec, 52°C for 3 min, 72°C for 1 min and 30 sec (30 cycles), 72°C for 2 min (1 cycle)
			R-ACATTTTCCTCAAAGAACAT		
5		*S. aureus*	F-CTTAAATCTACMTATTCWAAAMAYATGTT	257	
			R-TCARCGTCAAAWGTRAACTCT		
14		*E. faecium*	F-GAAAGYTTRGATAGYTTTGC	164	
			R-RTTTTGRCTTTCTTSYTTCA		
16		*S. aureus*	F-CAGGAAAACACTTCGTTTAT	592	
			R-CTTCTATATCACTATCATTGTCATT		
Group 4					
4		*Enterococcus faecium*	F-ACTATGTCGTTGAGTCTAATGACT	430	
			R-AGCAAGATAGAATATTTACTTTTAAGTTT		
10b		*S. aureus*	F-TAAATAAAGACTCAGGAGAAGTA	200	
			R-TAGCAAGTTCTCGAACTGTT		
11		*E. faecalis*	F-TCTAGAATGCGTAAAAAGG	500	
			R-CCTTTGAAGATWGCRGTWAG		
12		*B. thuringiensis*	F-GAGCCTATAACAGAGTACACA	470	
			R-CAAATATAGGCTTTGTAGTTC		

### Extraction and confirmation of *Staphylococcus aureus* plasmids

Extraction of plasmids was carried out on all confirmed *S. aureus* isolates using the NucleoSpin miniprep kit (Macherey-Nagel, Germany), in accordance with the manufacturer’s instructions, with certain modifications. These modifications included the addition of 5 µL of 1 mg/mL lysostaphin (Fujifilm, Japan). The presence of plasmid DNA was verified through gel electrophoresis using a 1% (m/v) SeaKem^®^ agarose gel (Lonza, Switzerland), stained with 5 µL ethidium bromide (10 µg/mL) (Promega, USA) and run at 90 V for 2 h in 1X TBE (Merck, Germany) buffer (40 mM Tris-HCl, 20 mM NaOAc, and 1 mM EDTA, pH 8.5) (Merck, Germany). To improve the detection of large or low-copy-number plasmids that could be potentially missed, plasmid DNA from the isolates was re-run at a lower voltage (60 V) for a longer duration (2.5 h). A 1 kb ladder (Labnet, USA) served as a size reference for result interpretation, and the Biorad gel DocTM EZ (BioRad, USA) system was utilised to visualise the plasmids under ultraviolet (UV) light.

### Multiplex-PCR assays for identifying *Staphylococcus aureus* virulence genes

Two M-PCR assays were utilised to screen for the following virulence genes: (i) biofilm formation: clumping factor A (*clfA*) and B (*clfB*) (Cao et al. 2023); (ii) toxin production: exfoliative toxin A (*eta*) (Budzy´nska et al. 2021) and exfoliative toxin B (*etb*) (Mohseni et al. 2018); and (iii) haemolysin production: alpha haemolysin (*hla*), gamma haemolysin (*hlg*) and gamma haemolysin CB (*HlgCB*) (Cao et al. 2023) on plasmid-positive isolates. Previously characterised in-house *S. aureus* strains from the Department of Microbiology, UP culture bank served as positive controls. Where positive controls were unavailable, the amplified products were compared to published gene sizes (Budzy´nska et al. 2021; Cao et al. 2023). The primer sequences and M-PCR amplification conditions for the virulence gene M-PCR assays are detailed in Table 1. All primers were synthesised by Inqaba Biotechnical Industries (Pty) Ltd, South Africa. Clinically relevant virulence genes, including Panton–Valentine leukocidin (*lukF-PV*/*lukS-PV*) and toxic shock syndrome toxin (*tst*), were not included in the PCR assays, as this study focused on a selected panel of plasmid-associated virulence determinants. These genes were, however, assessed during WGS analysis.

The M-PCR assays for the identification, PBRT and detection of virulence genes in *S. aureus* isolates were performed in a total reaction volume of 13 µL per tube. Each reaction mixture contained 2.5 µL of 5X CelTaq buffer (final concentration 1X) (CelTaq™, Cellscript, USA), 0.3 µL of CelTaq DNA polymerase (5 U/µL) (CelTaq™, Cellscript, USA), 1 µL of a 10X primer mix (2 µM of each primer, final concentration 0.2 µM), 7.4 µL of NFW (Promega, USA) and 1.5 µL of template DNA (approximately 1 µg per 50 µL). The amplified products were compared to published gene sizes, with NFW (Promega, USA) serving as the negative control. The amplification process was conducted in a SimpliAmpTM thermocycler (Applied Biosystems, USA).

### Agarose gel electrophoresis for the detection of M-PCR assay amplicons

All amplified products resulting from the M-PCR and PBRT assays were visualised using a 1.5% (m/v) SeaKem^®^ agarose gel (Lonza, Switzerland) and stained with 5 µL of ethidium bromide (10 µg/mL) (Promega, USA). Gel electrophoresis was conducted using a buffer consisting of 1x Tris-Borate-Ethylenediaminetetraacetic acid (EDTA) (TBE) (Merck, Germany) buffer (40 mM Tris-hydrochloric acid (HCl), 20 mM sodium acetate (NaOAc), and 1 mM EDTA, pH 8.5) and allowed to run at 90 V for 1.5 h. The molecular weights of the amplification products were compared to the expected amplicon sizes using a 100 bp (Labnet, USA) and a 1 kb plus ladder (Labnet, USA) as size references. The Biorad gel DocTM EZ (BioRad, USA) system was utilised to visualise all amplicons under UV light.

### Whole genome sequencing of selected *Staphylococcus aureus* isolates

Ten representative *S. aureus* isolates were selected for WGS due to resource constraints and were chosen based on the presence and number of plasmids and *rep* genes per isolate, as well as the AST profiles of the isolates. Details of the selected *S. aureus* isolates are shown in Table 3. Virulence gene characteristics were excluded from the selection criteria due to the high similarity in virulence gene patterns exhibited by the study isolates. The selected *S. aureus* isolates were incubated overnight in BHI broth (LabM Limited, United Kingdom) at 37 °C in a shaking incubator (Stuart Orbital, UK) set to 180 rpm. Total genomic DNA was extracted from each sample using a commercial kit [ZR Fungal/Bacterial DNA MiniPrep™ (Zymo Research, USA)], following the manufacturer’s instructions. The NanoDropTM 1000 spectrophotometer (Thermo Fisher Scientific, USA) was utilised to measure the concentrations and purity [≥ 1.8 at absorbance ratios (A260/A280)] of the extracted genomic DNA. Horizontal gene transfer was not experimentally assessed in this study, the presence of plasmid-associated antibiotic resistance genes was inferred from molecular and genomic analyses.

**Table 3:** Whole genome sequencing selection criteria of *Staphylococcus aureus* isolates

Isolate	Antibiotic resistance profile	Number of plasmids	*rep* genes
SaS11	-	2	*rep16*, *rep10*, *rep16*
SaM38	cloxacillin, ceftaroline, ciprofloxacin, clindamycin, erythromycin	0	-
SaS49	-	2	*rep5a*, *rep13*, *rep16*
SaM59	cloxacillin, ciprofloxacin, erythromycin and tetracycline	1	*rep10*
SaM62	cloxacillin, gentamicin, ciprofloxacin, clindamycin and erythromycin	1	*rep10*, *rep13*
SaS71	-	3	*rep5a*, *rep16*
SaS73	Cotrimoxazole	2	*rep5a*, *rep16*
SaS81	clindamycin, erythromycin, Cotrimoxazole, tetracycline	4	*rep5a*, *rep7a*, *rep10a *, *rep16*
SaS92	-	1	-
SaS96	-	4	*rep5a*, *rep7a*, *rep10a*, *rep16*

WGS was conducted at the National Institute for Communicable Diseases (NICD), South Africa, using the Illumina NextSeq 2000 (Illumina Inc., USA) platform. The Nextera XT library preparation kit (Illumina Inc, USA) was utilised to prepare paired-end libraries, and sequencing was performed at a 2 × 150 bp read length with 100x coverage. Raw sequencing reads were processed through the Jekesa pipeline version 1.0 (https://github.com/stanikae/jekesa). In summary, paired-end reads were filtered using Trim Galore version 0.4.5.1 (https://github.com/FelixKrueger/TrimGalore) (Krueger et al. 2023) with quality scores above 30 and lengths exceeding 50 bp. SPAdes version 3.15.4 (https://github.com/ablab/spades?tab=readme-ov-file) was employed for *de novo* assembly and the resulting contigs were refined with Shovill version 1.1.0 (https://github.com/tseemann/shovill) (Nurk et al. 2017). Multilocus sequence typing (MLST) profiles were assigned using the MLST tool version 2.16.4 (https://github.com/tseemann/mlst) (Jolley and Maiden 2010). Assembly metrics were assessed using QUAST version 5.0.2 (http://quast.sourceforge.net/quast) (Gurevich et al. 2013). Whole-genome single-nucleotide polymorphism (SNP) variations were characterised using the SKA toolkit (https://github.com/simonrharris/SKA) (Harris 2018), with a SNP threshold of less than 20. Antibiotic resistance profiles and virulence genes were predicted using ResFinder 4.1 (http://genepi.food.dtu.dk/resfinder) (Clausen et al. 2018; Bortolaia et al. 2020), PointFinder 2.2.6 (https://bitbucket.org/genomicepidemiology/pointfinder_db/src/master/) (Zankari et al. 2012, 2017), VirulenceFinder 2.0 (CGE, Denmark) (https://cge.food.dtu.dk/services/VirulenceFinder/) (Camacho et al. 2009; Clausen et al. 2018) and AMRFinderPlus (https://github.com/ncbi/amr/wiki/Running-AMRFinderPlus) (Feldgarden et al. 2021).

Publicly available curated online databases, such as PubMLST (University of Oxford, UK) (Jolley et al. 2018) (https://pubmlst.org/) and Pathogen Watch (Wellcome Sanger Institute, UK) (https://pathogen.watch/), were utilised to confirm species identification and determine sequence types and clonal complexes. The Center for Genomic Epidemiology (CGE) database tool PlasmidFinder 2.1 (https://cge.food.dtu.dk/services/PlasmidFinder/) (Camacho et al. 2009; Carattoli et al. 2014) was employed to detect plasmids. *S. aureus* strain typing was conducted using CGE tools such as spaTyper 1.0 (https://cge.food.dtu.dk/services/spaTyper/) (Bartels et al. 2014) to ascertain spa types. The SCC*mec* types among MRSA isolates were characterised using SCC*mec*Finder 1.2 (https://cge.food.dtu.dk/services/SCCmecFinder/) (Kaya et al. 2018), and MGEs were identified using MGEFinder 1.0.6 (https://cge.food.dtu.dk/services/MobileElementFinder/) (Johansson et al. 2021). Additionally, the Resistance Gene Identifier (RGI) (Comprehensive Antibiotic Resistance Database (CARD), Canada) (https://card.mcmaster.ca/) (Alcock et al. 2023) was employed for ARG identification, while the VF Analyzer (Chinese Academy of Medical Sciences and Peking Union Medical College, China) (https://www.mgc.ac.cn/cgi-bin/VFs/v5/main.cgi) was used to detect virulence genes (Chen et al. 2005). Multiple tools were utilised for WGS data analysis to ensure that no genes were overlooked, as certain publicly available curated online databases may have lacked specific genes.

### Statistical analysis

The Analysis ToolPak add-in for Microsoft^®^ Excel^®^ (Microsoft Corporation, USA) was utilised to describe continuous variables, such as patient ages, which were summarised using means and medians. The statistical significance of differences in AST, plasmid typing, PBRT, and M-PCR results were assessed with the Fisher’s exact test while, the chi-square test examined the association between plasmid size and various *rep* genes. Pearson correlation was conducted to determine the relationships among antibiotic resistance, plasmids, *rep* genes, and virulence genes. All analyses were performed using STATA18 (StataCorp, USA), with *P* values < 0.05 (two-tailed) deemed statistically significant.

## Results

### Patients demographics

A total of 100 non-repetitive invasive *S. aureus* isolates [15% (15/100) MRSA and 85% (85/100) MSSA] were collected for this study. A total of 65% (65/100) of isolates were from male and 35% (35/100) from female patients. The ages of patients ranged from 6 months to 88 years, with a median age of 57 years (interquartile range: 35.5 years to 71 years). Most of the *S. aureus* isolates were collected from blood [51% (51/100)] and tissue [42% (42/100)] specimens, whilst fluid [4% (4/100)], bone [1% (1/100)], port tip [1% (1/100)] and pus [1% (1/100)] specimens were less common. The specimens were sourced from hospitals [96% (96/100)] and clinics [4% (4/100)] in Gauteng, South Africa. Among the isolates from inpatients, 64.6% (62/96) were from the general ward, 34.4% (33/96) were from the intensive care unit and 1% (1/96) were from a burn unit.

### Antibiotic resistance profiles of *Staphylococcus aureus* isolates

**Fig. 1:**
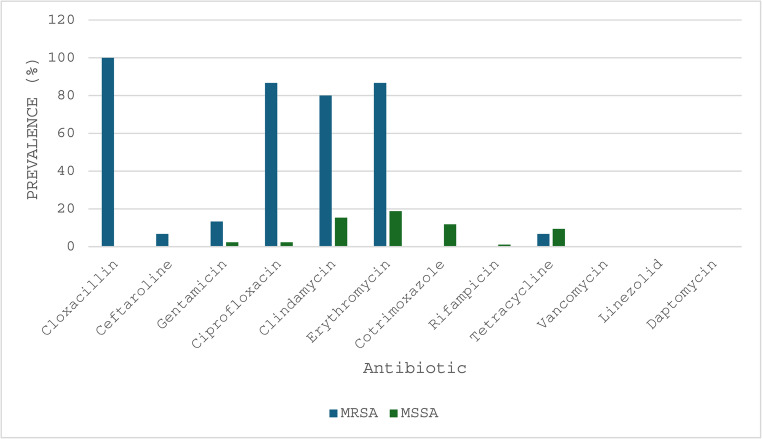
Prevalence of phenotypic antibiotic resistance among *Staphylococcus aureus* isolates. The figure illustrates the percentage of antibiotic resistance among MRSA (*n* = 15) and MSSA (*n* = 85) isolates. Resistance was assessed for multiple antibiotics using the VITEK® 2 automated system (bioMérieux, France). Resistance to cloxacillin was used as a proxy for methicillin resistance, indicating the presence of MRSA isolates.Abbreviations: MRSA: methicillin-resistant *Staphylococcus aureus*; MSSA: methicillin-susceptible *Staphylococcus aureus*

The MSSA and MRSA isolates demonstrated resistance to erythromycin at a rate of 29% (29/100) [MRSA: 86.7% (13/15); MSSA: 18.8% (16/85)], clindamycin at 26% (26/100) [MRSA: 80% (12/15); MSSA: 16.5% (14/85)], ciprofloxacin at 15% (15/100) [MRSA: 80% (12/15); MSSA: 3.5% (3/85)] and cotrimoxazole at 10% (10/100) [MRSA: 0% (0/15); MSSA: 11.8% (10/85)]. Resistance to the remaining antibiotics occurred at a frequency of less than 10% (detailed in Fig. 1). Notably, only 15% (15/100) of the isolates exhibited resistance to cloxacillin, which coincided with the presence of the *mecA* gene, thus defining these isolates as MRSA. All *S. aureus* isolates were susceptible to daptomycin, linezolid and vancomycin. Inducible clindamycin resistance was not assessed in this study, as D-testing was not performed; therefore, the prevalence of erythromycin-inducible clindamycin resistance could not be determined.

### Distribution of plasmids, antibiotic resistance profiles and virulence genes characteristics of plasmid-positive *Staphylococcus aureus*

Plasmids were detected by gel electrophoresis in 60% (60/100) of *S. aureus* isolates, comprising 83.3% (50/60) MSSA and 16.7% (10/60) MRSA. Plasmid prevalence did not differ significantly between MSSA [58.8% (50/85)] and MRSA [66.7% (10/15)] (*p* = 0.78). Plasmid sizes ranged from 0.85 kb to > 10 kb, with one to four plasmids per isolate. Two plasmids per isolate were found in 28% (28/100) of isolates [96.4% (27/28) MSSA] and ≥ 3 plasmids per isolate were found in 11% (11/100) of the isolates, all of which were MSSA. The MSSA isolates harboured significantly more plasmids than the MRSA isolates (*p* = 0.03).

Phenotypic antibiotic resistance was observed in 36.7% (22/60) of plasmid-positive isolates compared to 50% (20/40) of plasmid-negative isolates (*p* = 0.22). Among plasmid-positive MSSA isolates, 24% (12/50) were resistant to ≥ 1 antibiotic, while 76% (38/50) were susceptible to all tested antibiotics, with 71.1% (27/38) carrying ≥ 2 plasmids. In contrast, 80% (8/10) of plasmid-positive MRSA isolates were resistant to ≥ 4 antibiotics, all carrying a single plasmid. A very weak non-significant negative correlation was found between antibiotic resistance and plasmid number per isolate (*r* = -0.168, *p* = 0.931).

Among plasmid-positive isolates, the *hla* and *hlgCB* genes were detected in 98.3% (59/60) of the isolates, *clfA* in 96.7% (58/60) and *etb* in 95% (57/60). The *eta* gene was found in only 3.3% (2/60), both were MSSA isolates. Most isolates (90.9%; 54/60) carried the virulence profile: *clfA*, *etb*, *hlgCB* and *hla*. The number of virulence genes per isolate showed a very weak, non-significant positive correlation with plasmid number per isolate (*r* = 0.047, *p* = 0.719).

### Distribution of plasmid replicon genes among *Staphylococcus aureus* isolates

**Fig. 2:**
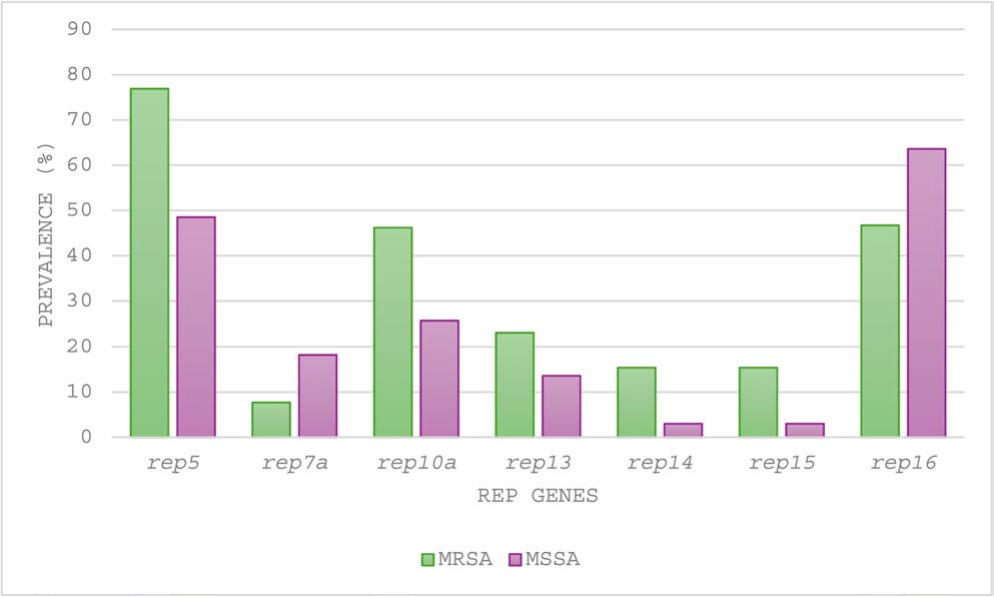
Prevalence of selected *rep* genes detected by PCR-based replicon typing among *Staphylococcus aureus* isolates. The figure illustrates the distribution of selected *rep* genes (*rep5a*, *rep7a*, *rep10a*, *rep13*, *rep14*, *rep15*, and *rep16*) detected by PBRT among MRSA (n = 15) and MSSA (n = 85) isolates. Abbreviations: MRSA: methicillin-resistant *Staphylococcus aureus*; MSSA: methicillin-susceptible *Staphylococcus aureus*; *rep*: replication initiation gene

Thirteen of the 20 *rep* genes screened (*rep1–rep4*, *rep6*, *rep7b*, *rep8*, *rep9*, *rep10b*, *rep11*, *rep12*, *rep17*, *rep18*) were absent. The *rep* genes were detected in 79% (79/100) of the *S. aureus* isolates by PBRT (Fig. 2). Among *rep*-positive isolates, 40.5% (32/79) carried a single *rep* gene, most commonly *rep16* [46.9% (15/32)], *rep10a* [21.8% (7/32)], and *rep5a* [12.5% (4/32)], while *rep7a*, *rep13* and *rep15* each occurred in 6.3% (2/32) of the isolates.

In plasmid-positive isolates (*n* = 60), the distribution was: *rep16* [65% (39/60)], *rep5a* [56.7% (34/60)], *rep10a* [23.3% (14/60)], *rep7a* [15% (9/60)], *rep13* [8.33% (5/60)], *rep14* [6.6% (4/60)] and *rep15* [3.3% (2/60)]. Notably, 10% (6/60) of plasmid-positive isolates had no *rep* genes and 12% (12/100) lacked both plasmids and *rep* genes. Conversely, 24% (24/100) of the isolates carried *rep* genes without plasmids, with half [50% (12/24)] having ≥ 2 *rep* genes. Plasmid sizes did not differ significantly by *rep* gene (χ² (14) = 10.84, *p* = 0.7), but the number of *rep* genes correlated weakly yet significantly with plasmid count per isolate (*r* = 0.315, *p* = 0.014).

### Association between replicon genes, antibiotic resistance and virulence genes

Among *rep*-positive *S. aureus* isolates (*n* = 79), 46.8% (37/79) were phenotypically resistant to at least one antibiotic. The most common *rep* genes in resistant isolates were *rep5a* [67.6% (25/37)], *rep16* [51.3% (19/37)] and *rep10a* [40.5% (15/37)] (supplementary Table S1). Most isolates [82.3% (65/79)] carried four virulence genes, while 7.6% (6/79) of the isolates had three, 6.3% (5/79) of the study isolates had five and 3.8% (3/79) had two. A very weak positive correlation was observed between *rep* gene count and virulence gene count (*r* = 0.171, *p* = 0.132).

### Whole genome sequencing analysis of the *Staphylococcus aureus* isolates

**Fig. 3:**
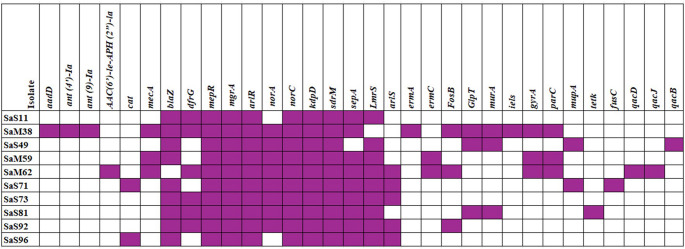
Antibiotic resistance genes detected by whole-genome sequencing in selected *Staphylococcus aureus* isolates. The heatmap displays the presence or absence of antibiotic-resistance genes in selected *S. aureus* isolates, as identified by whole-genome sequencing. Purple squares indicate the presence of a gene, while white squares indicate its absence.

WGS confirmed all ten isolates as *S. aureus* with no contamination, comprising 70% (7/10) MSSA and 30% (3/10) MRSA, consistent with ID-PCR. The *mecA* gene was present in all MRSA isolates. The most common ARG was *blaZ* [90% (9/10)], followed by *vanT* gene in the *vanG* cluster [80% (8/10)]. Notably, isolate SaM59 was phenotypically tetracycline-resistant despite lacking known tetracycline resistance genes. Full ARG profiles are shown in Fig. 3.

**Fig. 4:**
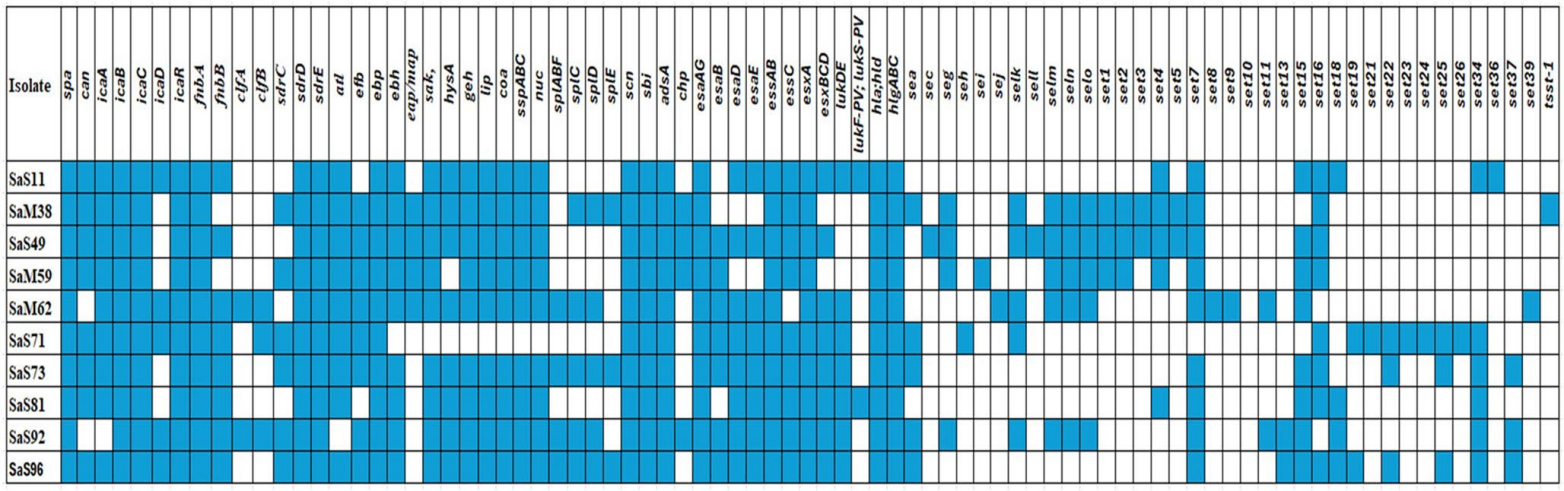
Virulence genes detected by whole-genome sequencing in selected *Staphylococcus aureus* isolates. The heatmap presents the presence or absence of virulence genes in selected *S. aureus *isolates, as determined by whole-genome sequencing. Blue squares indicate the presence of a gene, while white squares indicate its absence

Virulence gene profiling (Fig. 4) revealed widespread carriage of adhesion, enzyme, and toxin genes. All isolates carried *spa*, *icaBCR*, fnbA, *sdrDE* and *ebp* genes, with varying prevalence of other adhesion factors, leukotoxins and haemolysins. The PVL genes (*lukFS-PV*) were detected in 20% (2/10) of the isolates, which were both MSSA isolates. No exfoliative toxins were identified.

Several MGEs were identified in the *S. aureus* isolates, including composite transposons, insertion sequences (IS), plasmids and SCC*mec* elements. All three MRSA isolates carried distinct SCC*mec* types: type II (2 A) in SaM38, type IV (2B) in SaM59 and type V (5(2)) in SaM68. The IS elements were detected in 60% (6/10) of isolates, including all MRSA and three MSSA isolates. Detailed MGE data are in Table 4.

**Table 4:** A summary of the whole genome sequencing results of the selected *Staphylococcus aureus* isolates

Isolate	MSSA/MRSA	Sequence type (clonal complex)	*spa* type	SCC*mec* type	Mobile genetic elements
SaS11	MSSA	ST152	t355	-	Plasmid: *rep5a*(pN315, 24 653 bp); *rep16*(pSaaS6159, 20 730 bp)
SaM38	MRSA	ST36 (30)	t12	SCC *mec*_type_II(2A)	Plasmid:* rep5a* (pN315, 24 653 bp); *rep 16*(pSJH101, 3 429 bp); *rep16*(pWBG759, 28 384 bp); *rep22*(pAMalpha, 9 579 bp);* rep19*(SAP019A, 27 435 bp) Insertion sequence: ISSau6, ISSau5, ISSau4, ISSau2
SaS49	MSSA	ST45 (45)	t7022	-	Plasmid: *rep5a*(pN315, 24 653 bp); *rep16*(pSaaS6159, 20 730 bp); *rep20*(p11819p97, 22 317) Insertion sequence: ISSau3
SaM59	MRSA	ST22 (22)	t718	SCC *mec*_type_IV(2B)	Plasmid: *rep10a*(pDLK1, 2 402 bp); Insertion sequence: ISSau2, ISSau5, cn_24027_ISSau2
SaM62	MRSA	ST5 (5)	t9228	SCC *mec*_type_V (5(2))	Plasmid: *rep10a*(pDLK1, 2 402 bp); *rep13*(pSK41, 46 445 bp), Insertion sequence: ISSau5
SaS71	MSSA	ST1(1)	t127	-	Plasmid: *rep5a*(pN315, 24 653 bp); *rep16*(pSaS, 20 652 bp); *rep7a *(pTZ4, 4 555 bp);* rep7c*(MSSA476, 2 799 802 bp)
SaS73	MSSA	-	t701	-	Plasmid:* rep5a*(pN315, 24 653 bp); *rep16*(pSaaS6159, 20 730 bp)
SaS81	MSSA	ST152	t335	-	Plasmid: Plasmid: *rep5a*(pN315, 24 653 bp); *rep16*(pSaaS6159, 20 730 bp); *rep7a*(pS0385p1, 5 246 bp); *rep10a*(pDLK1, 2 402 bp)
SaS92	MSSA	ST5(5)	t548	-	Plasmid: *rep20*(pTW20, 29 585); insertion sequence: ISSau6 (3), cn_3976_ISSau6, cn_2258_ISSau6
SaS96	MSSA	ST6(5)	t701	-	Plasmid: *rep5a*(pN315, 24 653 bp); *rep16*(pSaaS6159, 20 730 bp); *rep7a*(pTZ4, 4 555 bp); Insertion sequence: ISSau3

WGS revealed that all representative *S. aureus* isolates carried plasmids, with the most common plasmid being pN315 (24 653 bp) at 70% (7/10), followed by pSaaS6159 (20 730 bp) at 50% (5/10), pDLK1 (2 402 bp) at 30% (3/10) and pTZ4 (4 555 bp) at 20% (2/10). Other plasmids detected included: SAP019A, pSJH101, pWBG759, pAMalpha, pSAS, pS0385P1, p11819P97, pSK41 and pTW20 each identified in different isolates. Several plasmid *rep* gene families were identified, with Rep_3 (*rep5a*) and Inc18 (*rep16*) being the most prevalent, co-occurring in 70% (7/10) of isolates. The *rep5a* gene, found on pN315 plasmids, was linked to *blaZ* (penicillin), *qacA* (antiseptics) and *mupA* (mupirocin) genes, while the Inc18 (*rep16*) gene, present on plasmids pSaaS6159, pSAS, pSJH101 and pWBG759, was associated with the *blaZ* and *qacB* genes, though some Inc18 plasmids lacked ARGs. Other *rep* families carried distinct resistance genes: Rep_trans (*rep7a*) in plasmid pTZ4 and pS0385p1 harboured the *tetK* (tetracycline) and *cat* (chloramphenicol) genes; The RepL (*rep10a*) gene in plasmid pDLK1 carried the *ermC* gene (macrolide-lincosamide-streptogramin resistance); Rep1 (*rep13*) gene in plasmid pSK41 carried the *qacD* gene; RepA_N (*rep20*) gene in plasmid p11819p97 and pTW20 carried the *blaZ* and *mupA* gene; RepA_N (*rep19*) gene in plasmid SAP019A lacked ARGs; The Rep1 (*rep22*) gene in plasmid pAMalpha1 carried the *aadD* and *bleo* genes (aminoglycoside resistance) and Rep_trans (*rep7c*) in plasmid MSSA476 carried *fusc* gene (fusidic acid resistance). Some discrepancies were noted between plasmid gel electrophoresis and WGS. WGS detected a higher number of plasmids than gel electrophoresis in isolates SaS11 (2 vs. 1), SaM38 (5 vs. 0), SaM62 (2 vs. 1), and SaS71 (4 vs. 3), whereas gel electrophoresis detected more plasmids than WGS in isolate SaS96 (4 vs. 3).

WGS identified multiple sequence types (STs) among the isolates, comprising both MRSA and MSSA lineages. The MRSA isolates included ST22 (SaM59, spa type t718), ST35 (SaM38, spa type t12) and ST5 (SaM62, spa type t9228), while MSSA isolates comprised ST152, ST5, ST6, ST1 and ST45. The ST152 and ST5 were each detected in two isolates: ST152 in SaS11 and SaS81 (Pretoria) with spa types t355 and t335, respectively, and ST5 in SaM62 (Johannesburg) and SaS92 (Pretoria) with spa types t9228 and t548, respectively. Two MSSA isolates were identified as ST6 (SaS73 and SaS96, spa type t701). Single MSSA isolates were assigned to ST1 (SaS71, t127) and ST45 (SaS49, t7022). Detailed SCC*mec*, ST and spa type combinations are provided in Table 4.

## Discussion

This study examined antibiotic resistance in *S. aureus*, with a particular focus on plasmid diversity and the relationship between *rep* genes and antibiotic resistance profiles. Elderly patients (above 60 years) accounted for the majority of the *S. aureus* isolates in this study. This observation is likely multifactorial and may be attributed to the increased prevalence of comorbid conditions, reduced immune function, and more frequent healthcare exposure associated with advancing age. This trend is consistent with studies from Asia (Hasmukharay et al. 2023), Europe (Thorlacius-Ussing et al. 2019) and Oceania (Williamson et al. 2014b), which also reported increased susceptibility in older age groups. The predominance of male patients observed in this study is consistent with previous reports (Williamson et al., 2014a; Thorlacius-Ussing et al. 2019) and may be explained by a combination of biological, behavioural and environmental factors, including differences in occupational exposure, healthcare-seeking behaviour and hygiene practices (Humphreys et al. 2015).

The prevalence of MRSA in this study was 15% (15/100), which aligns with previous reports from South Africa (Oosthuysen et al. 2014; Segal et al. 2023) and suggests relatively stable MRSA rates in private healthcare settings. While this prevalence is lower than that reported in some global regions (Saenhom et al. 2022), the clinical significance remains substantial due to the high proportion of MDR isolates among MRSA strains. In this study, 66.7% (10/15) of MRSA isolates were MDR, defined as resistance to at least one antibiotic in three or more classes. This finding highlights the continued therapeutic challenges posed by MRSA infections, particularly in clinical environments where treatment options may be limited.

Encouragingly, no phenotypic resistance to vancomycin, linezolid or daptomycin was detected among the isolates, indicating that these last-resort antibiotics remain effective for the treatment of *S. aureus* infections, including MDR-MRSA. However, the detection of the *vanT* gene in a substantial proportion of isolates [70% (7/10)] by WGS indicates the potential for low-level vancomycin resistance in these isolates, as *vanT* contributes to altered cell wall precursors via the vancomycin-resistance cassettes *vanC*, *vanE*, *vanG* and *vanN* (Aslanimehr et al. 2024). Furthermore, the detection of ceftaroline resistance in an MDR-MRSA isolate is notable, as ceftaroline is a newer anti-MRSA agent designed to target resistant strains (Duplessis and Crum-Cianflone 2011; Torres et al. 2023). This finding may signal the early emergence of reduced susceptibility to newer antimicrobial agents and underscores the importance of continued monitoring. Furthermore, the high prevalence of the *blaZ* gene reinforces the enduring challenge of β-lactam resistance in *S. aureus* (Hnini et al. 2024). Discrepancies between phenotypic tetracycline resistance and the absence of *tet* genes in some isolates may be explained by alternative mechanisms, such as multidrug efflux pumps that lower intracellular antibiotic concentrations (Zack et al. 2024).

The presence of key adhesion genes, including *spa*, *icaADBC* operon (*icaBCR*), fnbA, *sdrDE* and *ebp*, among all the selected isolates suggests a strong potential for biofilm formation. Biofilm formation is a well-recognised mechanism that enhances *S. aureus* survival under antimicrobial and environmental stress (Guo et al. 2022). The widespread detection of leukocidin genes, particularly *lukED*, aligns with epidemiological reports indicating the high prevalence of this gene in clinical isolates, including those associated with diabetic foot infections (Kananizadeh et al. 2019; Bennett and Thomsen 2020). The PVL genes (*lukFS-PV*) are typically linked to community-associated infections (Boswihi and Udo 2018). These genes were detected in two MSSA isolates, both ST152, a lineage typically linked to community-associated infections (Mzee et al. 2023). Both patients were hospitalised, but it is unclear whether the infections were community- or healthcare-acquired, indicating that community-associated lineages such as ST152 may also circulate in hospital settings.

The *etb* [95% (57/60)] and *eta* in [3.3% (2/60)] genes were detected in the plasmid-positive *S. aureus* isolates with M-PCR assays but were not detected with WGS. Similarly, the *clfA* gene was detected with PCR in all ten representatives, but only in two isolates by WGS. This discrepancy may be due to limitations in short-read sequencing and assembly, such as low read coverage in plasmid regions, sequence variation or fragmentation and filtering during contig assembly (Probst et al. 2021; Johnson et al. 2023). In this study, gene prediction was performed on assembled contigs rather than raw sequencing reads, which may have contributed to the non-detection of certain genes. The lack of association between replicon type and virulence gene carriage suggests that plasmid-borne traits in *S. aureus* are more strongly driven by antimicrobial selection than by virulence factors, with most virulence genes residing on the chromosome.

Plasmids were detected in 60% (60/100) of the isolates. While the overall prevalence of plasmids was not significantly different between MRSA and MSSA (*p* > 0.05), MSSA isolates carried more plasmids on average (*p* = 0.03). This may be linked to the higher frequency of MSSA isolates in this study, plasmid loss during extraction or the difficulty of detecting large plasmids (> 10 kb) by gel electrophoresis. Furthermore, isolate SaS96 (MSSA) showed four plasmids by gel electrophoresis but only three by WGS, suggesting either overestimation by gel electrophoresis or nonspecific bands. This highlights that while gel electrophoresis is cost-effective, this method may miss plasmids larger than 10 kb or those with low copy numbers.

WGS of the ten representative isolates revealed that all isolates carried plasmids of diverse types and sizes. The most prevalent plasmid, pN315 (24 653 bp), was identified in 70% (7/10) of isolates. This plasmid, first described in the *S. aureus* N315 strain (Kuroda et al. 2001), has also been reported in other bacterial genera, including *Enterococcus faecium*, suggesting its widespread distribution across different environments (Cho et al. 2020). Moreover, pN315 is widespread globally and has been associated with the Tn1546-like elements linked to vancomycin resistance dissemination in *E. faecium* (Simjee et al. 2002; Novais et al. 2008; Wang et al. 2022; Huang et al. 2024). Plasmid pSaa6159 (20 730 bp) was detected in 50% (5/10) of isolates, all of which were MSSA. This plasmid carried the *blaZ* gene, encoding β-lactamase and conferring resistance to penicillin. Its presence in MSSA isolates suggests that it may contribute to the dissemination of β-lactam resistance within *S. aureus* populations. The detection of this plasmid in strains not classified as MRSA highlights the potential for resistance determinants to circulate beyond traditionally resistant lineages.

The relationship between plasmid carriage and antibiotic resistance in *S. aureus* appears to be complex. In this study, no direct association was observed between the number of plasmids per isolate and the number of antibiotics to which the isolates were resistant. This suggests that resistance is influenced more by the presence of specific plasmids and genetic determinants rather than plasmid abundance alone. The relatively lower phenotypic resistance observed in plasmid-positive isolates may reflect the predominance of MSSA strains carrying plasmids encoding resistance to antibiotics not routinely included in AST panels, such as chloramphenicol, fusidic acid, mupirocin and penicillin. Penicillin was not included in the AST panel as routine susceptibility testing relies on cloxacillin as a surrogate for β-lactam resistance testing in this setting.

WGS of MRSA isolates (SaM38, SaM59 and SaM62) further indicated that not all resistance determinants were plasmid-encoded, suggesting that chromosomal mechanisms, such as mutations or efflux systems, may also contribute to multidrug resistance. The identification of key resistance genes, including *ermC* and *mupA*, on specific plasmids reinforces the importance of plasmid characterisation in understanding resistance profiles. Collectively, these findings indicate that antimicrobial resistance in *S. aureus* is driven by a combination of plasmid-mediated and chromosomal mechanisms.

The distribution of plasmid replicon types observed in this study reflects the heterogeneity of circulating *S. aureus* strains. The predominance of *rep16* [65% (39/60)] and *rep5a* [56.7% (34/60)] genes among plasmid-positive isolates suggests that these replicon types are well established within this population. The frequent co-occurrence of *rep5a* and *rep16* within isolates may indicate a selective advantage, potentially contributing to the persistence of these plasmids in clinical settings. In the present study, no isolate carried more than one *rep* gene per plasmid, in line with Snoussi et al. (2023), though multi-replicon plasmids have been reported in Malaysia by Al-Trad et al. (2023).

The association between *rep7a* plasmids and the *tetK* gene, which has been previously described (McCarthy and Lindsay 2012), was also observed in this study. In addition, the *rep7a* plasmid was found to carry the *cat* gene. Similar observations have been made in Malaysia, where the *cat* gene was found on both *rep7a* and *rep13* plasmids (Al-Trad et al. 2023). This highlights the flexibility of the *rep7a* plasmids in acquiring different resistance determinants. This supports the concept that certain plasmid types may exhibit flexibility in acquiring and maintaining different ARGs. The close association between plasmid pDLK1 carrying the *rep10a* gene and the *ermC* gene observed in this study reflects a recurring pattern in *S. aureus* populations. Similar co-location of the *ermC* gene with the *rep10a* gene has been documented on plasmid pE5 in Saudi Arabia (Snoussi et al. 2023) and on plasmid pNS2 in Malaysia (Al-Trad et al. 2023), suggesting that the association between the *ermC* gene and *rep10a* gene may be geographically widespread. While plasmids are not invariably linked to antibiotic resistance genes, the repeated occurrence of *ermC* within the *rep10a* plasmid supports the concept, outlined by McCarthy and Lindsay (2012), that certain *rep* genes have a strong and persistent association with specific antibiotic resistance genes. Such stable ARG–replicon associations may facilitate the maintenance and dissemination of antibiotic resistance traits within diverse *S. aureus* lineages.

In this study, the *rep16* gene was also associated with multiple resistance determinants, including *blaZ*, *mupA* and *qacA/B*. This suggests that *rep16*-containing plasmids may contribute to multidrug resistance phenotypes. The association of *rep5a* with *blaZ* on pN315 plasmids further supports the role of these plasmids in β-lactam resistance. In addition, less frequently detected replicon types, such as *rep20* and *rep22*, were associated with specific resistance determinants, including penicillin and aminoglycoside resistance. Although less prevalent, these replicon types may contribute to the overall diversity of resistance mechanisms within *S. aureus* populations.

The presence of the three SCC*mec* types (SCC*mec* type II (2 A), SCC*mec* type IV (2B) and SCC*mec* type V (5(2)) in MRSA isolates indicates the co-circulation of both hospital-associated (HA-MRSA) and community-associated (CA-MRSA) lineages within Gauteng private healthcare settings. Furthermore, the detection of multiple plasmids in the SCC*mec* type II isolate (SaM38) may reflect an increased capacity to accumulate resistance determinants, contributing to multidrug resistance phenotypes.

Several well-recognised *S. aureus* STs were identified in this study, reflecting both MRSA and MSSA lineages. The detection of PVL-positive ST152 (CC152) in two MSSA isolates (SaS11 and SaS81) with different spa types (t355 and t335), both originating from Pretoria, suggests possible local transmission with minor genetic variation. ST152 has been widely reported as a dominant lineage in Africa and has been identified in both human and animal hosts, indicating potential adaptability across different reservoirs (Mzee et al. 2023). Its presence in this study highlights its continued circulation in the region.

Globally recognised MRSA lineages were also identified, including ST22-MRSA-IV and ST35-MRSA-II. ST22-MRSA-IV, in particular, has been associated with resistance to fluoroquinolones and macrolides (Silva et al. 2022), which is consistent with the resistance patterns observed in this study. The identification of these lineages supports their continued presence in South Africa and underscores the importance of ongoing surveillance.

The detection of ST5 (CC5) in both MRSA and MSSA isolates reflects the versatility of this lineage, which is recognised as a globally distributed and successful clone (van Rensburg et al. 2010; Boswihi et al. 2022; Silva et al. 2022). The presence of both methicillin-resistant and methicillin-susceptible variants suggests the potential for acquisition or loss of resistance determinants within this lineage, contributing to its adaptability in different environments.

Community-associated MRSA lineages were also observed. The ST1-t127 lineage, historically associated with community-acquired infections (Namoune et al. 2023), has been reported in both human and animal contexts (Parisi et al. 2016; Kaiser-Thom et al. 2022). In this study, resistance determinants associated with this lineage, including those linked to fusidic acid (Williamson et al. 2014a) and mupirocin resistance (Carter et al. 2018), were also identified, highlighting the clinical relevance of this lineage.

Less frequently reported lineages, including ST45 (CC45) and ST6 (CC5), were also detected. ST45 is widely distributed internationally and has been associated with both MSSA and MRSA variants Effelsberg et al. 2020; Silva et al. 2022). Similarly, ST6 has been reported in association with foodborne outbreaks and community-associated infections (Liao et al. 2020; Sales et al. 2024). The presence of these lineages in this study further reflects the genetic diversity of *S. aureus* circulating within the study population.

The limitation of this study was the use of gel electrophoresis alone for plasmid screening, which could not detect plasmids > 10 kb. According to WGS data, the largest plasmid detected among the isolates was 46 445 bp in size. Additionally, inducible clindamycin resistance was not assessed, as D-testing was not included in the routine diagnostic workflow, which may have resulted in an underestimation of clindamycin resistance among erythromycin-resistant isolates. Future studies could incorporate PFGE methods using the Centres for Disease Control and Prevention (CDC) PulseNet protocols (McDougal et al. 2003; Marasini and Fakhr 2014) to detect larger plasmids of > 10 kb in size if WGS is not performed, as well as include D-testing to better characterise inducible resistance. Broader sampling across public and private healthcare settings is also recommended for a more representative national picture. Continued use of WGS will be essential for detailed surveillance, guiding infection control and tracking the molecular epidemiology of *S. aureus* in South Africa.

This study demonstrated an association between specific plasmid *rep* genes and antibiotic resistance genes in *S. aureus*, highlighting the role of plasmids in shaping antimicrobial resistance profiles in clinical isolates. Notably, *rep5*a (pN315) and *rep16* (pSaaS6159) were associated with *blaZ*, while *rep7a* and *rep10a* were linked to tetracycline, chloramphenicol and macrolide resistance genes, respectively. The predominance of MSSA isolates carrying plasmids encoding resistance to non-routinely tested antibiotics suggests potential underestimation of resistance in clinical settings. The identification of both MRSA and MSSA lineages, including diverse sequence types, underscores the genetic diversity and ongoing transmission of *S. aureus* in Gauteng. These findings highlight the importance of integrating plasmid typing and genomic approaches into surveillance programmes to better understand and monitor the dissemination of antibiotic resistance.

## Conclusion

This study established baseline information on the relationships among antibiotic resistance, plasmids, *rep* genes and virulence genes in *S. aureus* isolates from clinical settings in Gauteng, South Africa. Specific associations between plasmid replicon types and ARGs were identified, highlighting the contribution of plasmids to resistance in these isolates. The detection of both MRSA and MSSA lineages, together with evidence of local transmission, indicates ongoing circulation of genetically diverse strains within the province. These findings underscore the importance of continued genomic surveillance to monitor clonal spread and inform strategies aimed at limiting the dissemination of antibiotic resistance and potential outbreaks in clinical settings.

## Supplementary Information

Below is the link to the electronic supplementary material.

Supplementary Material 1

## Data Availability

The datasets generated during and/or analysed during the current study are available from the corresponding author upon request.
