# Supplementary material for: Plasmid diversity, antibiotic resistance and virulence genes associated with Staphylococcus aureus isolates from private hospitals in Gauteng, South Africa
Source: World J Microbiol Biotechnol. 2026 Apr 25;42(5):233. doi: 10.1007/s11274-026-04956-4 (PMC13109123; doi:10.1007/s11274-026-04956-4)
Supplement: Supplementary file 1 — Supplementary Material 1 [file 11274_2026_4956_MOESM1_ESM.docx]

**Supplementary Material for: Plasmid diversity, antibiotic resistance and virulence genes associated with *Staphylococcus aureus* isolates from private hospitals in Gauteng, South Africa**

**Table S1. Summary findings from plasmid typing, PBRT and AST profiles of the *Staphylococcus aureus* isolates**

| **Isolate** | **MSSA/MRSA** | ***rep* gene pattern** | **AST profile** | **Plasmid size (kb)** |
| --- | --- | --- | --- | --- |
| 1 | MSSA | *rep10a* | - | - |
| 2 | MSSA | *rep10a-rep15* | CLI-ERY | - |
| 3 | MSSA | *rep5-rep16* | CLI-ERY | - |
| 4 | MSSA | *rep16* |  | - |
| 5 | MSSA | *rep10a* | CLI-ERY | 1.8; 10 |
| 6 | MSSA | *rep10a-rep16* |  | - |
| 7 | MSSA | *rep7a-rep10a-rep15* | GEN | - |
| 10 | MSSA | *rep10a* | CLI-ERY-TET | - |
| 11 | MSSA | *rep5-rep16* | - | >10 |
| 12 | MSSA | *rep10a* | CLI-ERY-SXT |  |
| 13 | MRSA | *rep5-rep10a-rep13-rep14* | CLX-CIP-CLI-ERY | 1.4 |
| 14 | MSSA | *rep13* | - | - |
| 17 | MSSA | *rep7a* | - | - |
| 19 | MSSA | *rep5* | GEN | - |
| 20 | MSSA | *rep5-rep10a* | - | - |

Abbreviations: CLI, clindamycin; CLX, cloxacillin; CIP, ciprofloxacin; ERY, erythromycin; GEN, gentamicin; RIF, rifampicin; SXT, trimethoprim-sulfamethoxazole; TET, tetracycline

**Table S1. Summary findings from plasmid typing, PBRT and AST profiles of the *Staphylococcus aureus* isolates (continued)**

| **Isolate** | **MSSA/MRSA** | ***rep* gene pattern** | **AST profile** | **Plasmid size (kb)** |
| --- | --- | --- | --- | --- |
| 22 | MSSA | *rep5* | CLI-ERY | - |
| 25 | MSSA | *rep5-rep10a-rep15* | - | - |
| 26 | MSSA | *rep15* | - | - |
| 28 | MSSA | *rep7a-rep10a-rep16* | - | 1.2; 1.6; >10 |
| 29 | MSSA | *rep5-rep10a-rep16* | CLI-ERY | 1.4 |
| 30 | MRSA | *rep5-rep10a* | CLX-CIP-CLI-ERY | - |
| 31 | MSSA | *rep5-rep14* | - | >10 |
| 32 | MSSA | *rep7a* | - | 2.8; 2.9 |
| 33 | MSSA | *rep5-rep16* | - | >10; >10 |
| 34 | MSSA | *rep5-rep7a-rep16* | - | 2.7; >10;>10 |
| 35 | MSSA | *rep5-rep7a-rep14* | ERY | >10 |
| 36 | MSSA | *rep5-rep10a* | - | >10 |
| 37 | MSSA | *rep5a-rep10a* | SXT | >10; >10 |
| 38 | MRSA | *rep16* | CLX-CPT-CIP-CLI-ERY | - |
| 40 | MRSA | *rep5-rep10a-rep14-rep15* | CLX | >10 |
| 41 | MRSA | *rep5-rep7a-rep10a* | CLX-GEN-CIP-ERY | >10 |
| 42 | MSSA | *rep15* | - | >10 |

Abbreviations: CLI, clindamycin; CLX, cloxacillin; CIP, ciprofloxacin; ERY, erythromycin; GEN, gentamicin; RIF, rifampicin; SXT, trimethoprim-sulfamethoxazole; TET, tetracycline

**Table S1. Summary findings from plasmid typing, PBRT and AST profiles of the *Staphylococcus aureus* isolates (continued)**

| **Isolate** | **MSSA/MRSA** | ***rep* gene pattern** | **AST profile** | **Plasmid size (kb)** |
| --- | --- | --- | --- | --- |
| 45 | MSSA | *rep5-rep10a-rep16* | CLI-ERY | >10 |
| 46 | MSSA | *rep5-rep16* | - | >10; >10 |
| 47 | MSSA | *rep5* | - | 2; 8; 10 |
| 49 | MSSA | *rep5-rep13-rep16* | - | 1.8; >10 |
| 50 | MSSA | *rep5-rep13-rep16* | - | 1.8; >10 |
| 53 | MSSA | *rep7a-rep13* | - | >10 |
| 54 | MSSA | *rep5-rep16* | - | >10; >10 |
| 55 | MRSA | *rep5-rep16* | CLX-CIP-CLI-ERY | >10 |
| 56 | MRSA | *rep5-rep15-rep16* | CLX-CIP-CLI-ERY | - |
| 57 | MSSA | *rep16* | - | >10; >10 |
| 58 | MSSA | *rep5* | TET | >10; >10 |
| 59 | MRSA | *rep10* | CLX-CIP-CLI-ERY-TET | 0.85 |
| 60 | MSSA | *rep5-rep16* | - | >10 |
| 61 | MRSA | *rep5-rep16* | CLX-CIP-CLI-ERY | >10 |
| 62 | MRSA | *rep10-rep13* | CLX-GEN-CIP-CLI-ERY | >10 |
| 63 | MRSA | *rep5-rep16* | CLX | >10; >10 |
| 64 | MRSA | *rep5-rep13-rep16* | CLX-CIP-CLI-ERY | >10 |

Abbreviations: CLI, clindamycin; CLX, cloxacillin; CIP, ciprofloxacin; ERY, erythromycin; GEN, gentamicin; RIF, rifampicin; SXT, trimethoprim-sulfamethoxazole; TET, tetracycline

**Table S1. Summary findings from plasmid typing, PBRT and AST profiles of the *Staphylococcus aureus* isolates (continued)**

| **Isolate** | **MSSA/MRSA** | ***rep* gene pattern** | **AST profile** | **Plasmid size (kb)** |
| --- | --- | --- | --- | --- |
| 65 | MRSA | *rep5-rep16* | CLX-CIP-CLI-ERY | >10 |
| 66 | MSSA | *rep16* | - | >10; >10 |
| 67 | MSSA | *rep16* | - | >10; >10 |
| 68 | MSSA | *rep16* | - | >10 |
| 69 | MSSA | *rep5-rep16* | CLI-ERY | - |
| 70 | MSSA | *rep5-rep16* | - | >10; >10 |
| 71 | MSSA | *rep5-rep16* | - | 3.1; >10; >10 |
| 73 | MSSA | *rep5-rep16* | SXT | 6; >10 |
| 75 | MSSA | *rep13* | CLI-ERY | - |
| 77 | MSSA | *rep16* | - | >10; >10 |
| 78 | MSSA | *rep13-rep16* | CLI-ERY | - |
| 79 | MSSA | *rep16* | - | >10; >10 |
| 80 | MSSA | *rep16* | - | >10; >10; >10 |
| 81 | MSSA | *rep5-rep7a-rep10-rep16* | CLI-ERY-SXT-TET | 1.5; 5; >10; >10 |
| 83 | MSSA | *rep16* | CIP | - |
| 84 | MSSA | *rep5-rep16* | - | >10; >10 |
| 85 | MSSA | *rep16* | - | 1.3; >10; >10 |
| 86 | MSSA | *rep16* | CLI-ERY | >10; >10 |

Abbreviations: CLI, clindamycin; CLX, cloxacillin; CIP, ciprofloxacin; ERY, erythromycin; GEN, gentamicin; RIF, rifampicin; SXT, trimethoprim-sulfamethoxazole; TET, tetracycline

**Table S1. Summary findings from plasmid typing, PBRT and AST profiles of the *Staphylococcus aureus* isolates (continued)**

| **Isolate** | **MSSA/MRSA** | ***rep* gene pattern** | **AST profile** | **Plasmid size (kb)** |
| --- | --- | --- | --- | --- |
| 87 | MSSA | *rep16* | - | >10; >10 |
| 88 | MSSA | *rep7a-rep13* | - | - |
| 89 | MSSA | *rep16* | - | >10; >10 |
| 90 | MSSA | *rep14-rep16* | - | - |
| 91 | MSSA | *rep7a-rep13-rep16* | - | - |
| 94 | MSSA | *rep16* | - | >10; >10 |
| 95 | MSSA | *rep16* | - | >10; >10; >10 |
| 96 | MSSA | *rep5-rep7a-rep10a-rep16* | - | 5; >10; >10; >10 |
| 97 | MSSA | *rep5-rep10a-rep16* | CIP-CLI-ERY | 1.6; 2; 6 |
| 98 | MSSA | *rep5-rep7a-rep16* | RIF | 3; >10; >10 |
| 99 | MSSA | *rep5-rep16* | SXT | >10; >10 |
| 100 | MSSA | *rep5-rep16* | SXT | >10; >10 |

Abbreviations: CLI, clindamycin; CLX, cloxacillin; CIP, ciprofloxacin; ERY, erythromycin; GEN, gentamicin; RIF, rifampicin; SXT, trimethoprim-sulfamethoxazole; TET, tetracycline
